# Untargeted Phytochemical Profile, Antioxidant Capacity and Enzyme Inhibitory Activity of Cultivated and Wild Lupin Seeds from Tunisia

**DOI:** 10.3390/molecules26113452

**Published:** 2021-06-07

**Authors:** Amna Ben Hassine, Gabriele Rocchetti, Leilei Zhang, Biancamaria Senizza, Gökhan Zengin, Mohamad Fawzi Mahomoodally, Mossadok Ben-Attia, Youssef Rouphael, Luigi Lucini, Safia El-Bok

**Affiliations:** 1 Laboratory of Biodiversity, Biotechnologies and Climate Change (LR11/ES09), Department of Biology, Faculty of Sciences of Tunis, University of Tunis El-Manar, Tunis 2092, Tunisia; amnabenhassine@gmail.com (A.B.H.); selbok151@gmail.com or; 2 Department for Sustainable Food Process, Università Cattolica del Sacro Cuore, Via Emilia Parmense 84, 29122 Piacenza, Italy; gabriele.rocchetti@unicatt.it (G.R.); leilei.zhang@unicatt.it (L.Z.); biancamaria.senizza@unicatt.it (B.S.); 3 Physiology and Biochemistry Research Laboratory, Department of Biology, Science Faculty, Selcuk University, 42130 Konya, Turkey; gokhanzengin@selcuk.edu.tr; 4 Department of Health Sciences, Faculty of Medicine and Health Sciences, University of Mauritius, Réduit 230, Mauritius; f.mahomoodally@uom.ac.mu; 5 Environment Biomonitoring Laboratory (LR01/ES14), Bizerta Faculty of Sciences, University of Carthage, Zarzouna 7021, Tunisia; benattia.mossadok@gmail.com; 6 Department of Agricultural Sciences, University of Naples Federico II, 80055 Portici, Italy

**Keywords:** foodomics, untargeted profiling, radical scavenging, acetylcholine esterase inhibition, functional components, *Lupinus*, bioactive compounds, UHPLC-QTOF-MS

## Abstract

Lupin seeds can represent a valuable source of phenolics and other antioxidant compounds. In this work, a comprehensive analysis of the phytochemical profile was performed on seeds from three *Lupinus* species, including one cultivar (*Lupinus albus*) and two wild accessions (*Lupinus cossentinii* and *Lupinus luteus*), collected from the northern region of Tunisia. Untargeted metabolomic profiling allowed to identify 249 compounds, with a great abundance of phenolics and alkaloids. In this regard, the species *L. cossentinii* showed the highest phenolic content, being 6.54 mg/g DW, followed by *L. luteus* (1.60 mg/g DW) and *L. albus* (1.14 mg/g DW). The in vitro antioxidant capacity measured by the ABTS assay on seed extracts ranged from 4.67 to 17.58 mg trolox equivalents (TE)/g, recording the highest values for *L. albus* and the lowest for *L. luteus*. The DPPH radical scavenging activity ranged from 0.39 to 3.50 mg TE/g. FRAP values varied between 4.11 and 5.75 mg TE/g. CUPRAC values for lupin seeds ranged from 7.20 to 8.95 mg TE/g, recording the highest for *L. cossentinii*. The results of phosphomolybdenum assay and metal chelation showed similarity between the three species of *Lupinus*. The acetylcholinesterase (AChE) inhibition activity was detected in each methanolic extract analyzed with similar results. Regarding the butyrylcholinesterase (BChE) enzyme, it was weakly inhibited by the *Lupinus* extracts; in particular, the highest activity values were recorded for *L. albus* (1.74 mg GALAE/g). Overall, our results showed that *L. cossentinii* was the most abundant source of polyphenols, consisting mainly in tyrosol equivalents (5.82 mg/g DW). Finally, significant correlations were outlined between the phenolic compounds and the in vitro biological activity measured, particularly when considering flavones, phenolic acids and lower-molecular-weight phenolics.

## 1. Introduction

Increasing population, climatic conditions and reduction of fertile land for cultivation have led to hunger and malnutrition, thus justifying the need for scientists to find new and affordable food sources [1]. Accordingly, lupine is a crop growing on marginal agricultural land under a variety of environmental conditions, with great potential that deserves to be significantly added to existing food sources [2]. Only a few species, such as white lupine (*L. albus*), blue lupine (*L. angustifolius*) and yellow lupine (*L. luteus*), have been studied for their agronomic characteristics and nutritional values.

In most parts of the world, lupine has traditionally been used primarily for feeding livestock [3]. As a resource of plant proteins, lupine has seen a growing interest since its unique nutritional quality and health benefits became known. Lupine, a non-starchy grain legume, contains less fat (~6%), a high number of essential amino acids, important dietary minerals, higher levels of protein (~40%) and dietary fiber (~28%), which make it a good food ingredient [4]. Lupine is considered a cheap alternative to other legume crops, especially soybeans, as it contains comparable amounts of protein with a similar amino acid profile. Moreover, lupin species, i.e., *L. albus* and *L. angustifolius*, have low-fat content and a high content of dietary fibre [5] and, hence, are more palatable [6]. Phenolic compounds represent plant secondary metabolites, providing antioxidant, antiallergic, anti-inflammatory and anti-carcinogenic properties [7,8,9,10,11]. These properties generally protect against cellular damage and prevent the risk of degenerative diseases [12,13,14]. In this regard, seeds of the lupin species are a valuable source of phenolic compounds, such as flavones and isoflavones [15], commonly referred to as phytoestrogens, which are related to high antioxidant activity, although the presence of some amino acids and peptides can also contribute to this activity [16,17,18,19].

In the last years, enzyme inhibitory strategies have been considered as one of the most effective solutions to global health problems, including Alzheimer’s (AD) and diabetes mellitus (DM). Acetyl cholinesterase (AChE) is an important therapeutic target to alleviate the deterioration of cholinergic neurons in the brain and the loss of neurotransmission, i.e., one of the major causes of Alzheimer’s disease. In addition to AChE, butyrylcholinesterase (BChE) plays an important to alleviate the memory functions of AD patients [20]. Tyrosinase is a well-known copper-containing enzyme that catalyzes melanin biosynthesis and plays a crucial role in skin pigmentation disorders, such as melasma and age spots [21,22]. Furthermore, it can be considered as an anti-browning compounds in food and agriculture industries and as depigmentation agent in the cosmetic and medicinal industries [23]. Other important targeted enzymes are represented by alpha-amylase and glucosidase, two of the main enzymes in carbohydrate catabolism. Thus, the inhibition of their activity might contribute to manage blood glucose levels after a carbohydrate-rich diet [24]. However, synthetic drugs are known to have limitations due to short half-life and unfavorable side effects, such as gastrointestinal disturbances and hepatotoxicity. Therefore, an urgent need exists for alternative inhibitors from natural sources with minimal or no side effects.

Additionally, the presence of proteins and other functional molecules in species of *Lupinus* genus has gained significant interest among the scientific community to investigate various valuable fractions in different parts of the plant. For example, the seeds of lupin species with small amounts of toxic compounds have been considered safer for human and animal consumptions as they contain less anti-nutritional compounds than other leguminous plants, which significantly widened the spectrum of their potential application [16,17,18].

In order to investigate the comprehensive phytochemical composition of lupin seeds and other legumes, metabolomics-based studies focused on specific classes of compounds are gaining interest. For example, Llorach et al. [25] carried out a comparative metabolite fingerprinting of legumes using LC-MS-based untargeted metabolomics. The authors highlighted the applicability of metabolomics for evaluating the bioactive profile and the characteristic compounds of different legumes, including beans, chickpeas and lentils, promoting the utilization of metabolomics as a tool for the quality and authentication of different legumes.

Therefore, starting from the previous considerations, the aim of this study was to comparatively estimate the in vitro antioxidant and enzyme inhibitory properties of seeds of three Tunisian lupin species (two wild and one cultivated), also providing comprehensive information on their phytochemical profile, with a focus on beneficial health-promoting traits. The final aim is to advance the knowledge on the use of lupine in the production of health-promoting and functional foods.

## 2. Results and Discussion

### 2.1. Semi-Quantitative Analysis of Phenolics, Flavonoids and Anthocyanins Contents

The semi-quantitative analysis of the different phenolic classes was made by UHPLC-QTOF-MS, as shown in Table 1. Additionally, the in vitro antioxidant capacity (evaluated as DPPH, FRAP and CUPRAC values) results are shown in Table 2. Regarding the semi-quantitative analysis of the main classes of compounds detected in *Lupinus* species, the alkaloids class was found most abundant in two samples, namely *L. albus* and *L. luteus* (4.42 and 3.66 mg/g DW, respectively). In contrast, *L. cossentinii* showed the lowest content of total alkaloids (0.84 mg/g DW). Interestingly, a lower concentration of alkaloids was found in *L. cossentinii* species, although well-known to be the main constituents of a wide range of Lupinus species. The results on alkaloids contents are linked to those obtained for *L. albus* and *L. luteus* [26]. In reference to the anthocyanins, the highest amount was detected in the *L. luteus* with 0.26 mg/g DW, while the *L. albus* and *L. cossentinii* showed values of 0.11 and 0.13 mg/g DW, respectively. This is the first reported quantification of anthocyanins with UHPLC-QTOF-MS; therefore, our data are difficult to compare with existing literature, because no anthocyanins were previously found in the lupin seed coats by using the UHPLC-QTOF-MS technique.

Finally, regarding the remaining semi-quantitative values on phenolics, no significant differences were observed for other flavonoids and phenolic acids (Table 1), while *L. albus* was outlined as the richest (*p* < 0.05) source of lignans, being 0.26 mg/g DW. Moreover, *L. cossentinii* was found to be the richest source of other phenolics (quantified as tyrosol equivalents) at 5.82 mg/g DW.

Lupin seeds can induce, in certain cases, some side effects, which are related to alkaloids contents. According to literature, all lupin species contain alkaloids in different amounts. In fact, there are around 150 and up to 500 lupin species worldwide, but only seeds from few species have been used for human consumption (the so-called “edible lupins”), especially white lupin (*Lupinus albus* L.) and narrow-leaved lupin (*Lupinus angustifolius* L.). The seeds, as well as the leaves, of the lupin contain toxicologically relevant high alkaloids, depending on the botanical and geographical origin, as well as the soil composition and climate [27,28]. Our results suggested that the total alkaloids content in *L. albus* and in *L. luteus* are similar, considering that the seeds of these two species were collected in the same geographical region in Tabarka. Our findings on total alkaloids contents were closely linked to those obtained in Morocco for *L. albus* and *L. luteus*, collected in the same bioclimatic stage [29].

Furthermore, as part of the development of lupine crops, research is now directed towards the selection of low alkaloid content genotypes. However, lupine species with low alkaloid content can degenerate due to mutations that could lead to a decline in plant vigor. The reverse mutation is caused by crosses to bitter plants and mixed varieties, or in neighboring crops. Other authors further point out that the improvement in *L. luteus* focused on a single original mutation. Reproductive procedures involving other genetic loci for smoothness should be recommended not only for the production of double recessives as much as possible, but also to avoid toxic effects. For this purpose, a series of phenotypic properties could be used, for example the color of the seeds (white, brown, and sickle color), the characteristics of the hilum, the color of the flower and the color of the keel of the flower.

### 2.2. In Vitro Antioxidant Capacity

In order to test the in vitro antioxidant activities of the methanol extracts of *L. albus*, *L. luteus* and *L. cossentinii*, six different tests were used. The assays were radical scavenging (DPPH and ABTS), reducing power (CUPRAC and FRAP) and phosphomolybdenum and metal chelating (Table 2).

The in vitro antioxidant capacity measured by the ABTS method of the methanol seed extracts ranged from 17.58 to 4.67 mg TE/g, where the highest value was recorded for the *L. albus* and the lower for *L. luteus*. It has been previously reported that the antioxidant activity is positively related to the content of polyphenols [30,31]. In our studies, we found that the total phenolic content was highly correlated with ABTS activities, with a Pearson correlation coefficient of 0.948 (*p* < 0.01).

The DPPH radical was used to evaluate radical scavenging abilities of the tested lupin seed extracts. In our experimental conditions, the DPPH radical scavenging activity ranged from 0.39 up to 3.50 mg TE/g. These data are close to the results obtained by Siger et al. [31], reporting a DPPH radical scavenging activity from 0.88 to 2.03 mg TE/g in *L. albus* and *L. luteus*, respectively. In addition, our findings are similar to those reported by Dueñas et al. [21] for *Lupinus*, showing an increase from 170.3 to 690.2 mg TE/100 g on day 9 of germination.

FRAP values ranged between 4.11 and 5.75 mg TE/g (Table 2). The Pearson correlation analysis (with a significance level of *p* < 0.01) revealed a significant correlation between DPPH and FRAP values. In addition, Siger et al. [31] showed that the DPPH scavenging activity is well correlated with the total phenolic compounds content.

CUPRAC values for lupin seeds ranged from 7.20 to 8.95 mg TE/g, where the lowest value was for *L. albus*, and the highest was for *L. cossentinii*. As it was observed, CUPRAC and ABTS antioxidant capacity assay of lupin seed extracts showed higher values than DPPH and FRAP methods. Bojilov et al. [22] observed similar result for CUPRAC antioxidant capacity assay of lupin seed extracts.

The results of the phosphomolybdenum assay and the metal chelating showed a similarity among the three species of *Lupinus* under investigation. The three methanolic extracts showed results of 0.47, 0.44 and 0.41 mmol TE/g for *L. albus*, *L. cossentinii* and *L. luteus*, respectively. In addition, considering the lack in the literature of similar assay for the *Lupinu*s species, we found that Ohadoma and Eban [23] demonstrated that a higher phosphomolybdenum value also represents higher antioxidant activity, with a value of 525 mg AAE/g extract for *L. arboreus.* Because the authors used ascorbic acid as a standard, their results cannot compare with our presented results.

Finally, iron chelating activity values for *L. albus*, *L. cossentinii* and *L. luteus* were 3.38, 3.55 and 4.68 mg EDTAE/g, respectively. Our findings do not match with the work previously published by Ohadoma and Eban [23], in which the value of iron chelating activity was 35.33 mg ethylenediaminetetraacetic acid equivalents/g for *L. arboreus.* The different results could be explained by the differences in the chemical components in the *Lupinus* species or in soil composition. In addition, metal chelating ability is one minor role in the antioxidant mechanism of phenolics. Moreover, not only phenolics but also other phytochemicals could play role in the metal chelating activity. In fact, polysaccharides are reported to exhibit a strong chelating activity. When evaluating the results from the in vitro antioxidant capacity assays, the tested *Lupinus* extracts demonstrated antioxidant properties. Considering the side effects of synthetic antioxidants on human health, the *Lupinus* species could be considered as a potential source of natural antioxidants. However, we strongly suggested further in vivo experiments for supporting the observed antioxidant properties.

### 2.3. In Vitro Enzyme Inhibition Assays

To investigate the enzyme inhibitory capacity of the different *Lupinus* extracts, the cholinesterases AChE and BChE, together with the enzymes tyrosinase and α-amylase, were considered. The results obtained using the above-mentioned enzymes are illustrated in Table 3.

In the presented study, AChE inhibition activity was detected in each methanolic extract analyzed with the same results of 0.9, 0.93 and 1.04 mg galantamine equivalent (GALAE)/g for *L. albus*, *L. luteus* and *L. cossentinii*, respectively. Regarding the enzyme BChE, it exhibits the same low inhibition activity as the AChE for the *Lupinus* extract (Table 3). The highest activity values were recorded for *L. albus* (1.74 mg GALAE/g). Regarding tyrosinase inhibitory properties, all the extracts of the three *Lupinus* species have shown an important inhibitory activity against tyrosinase, with the highest activity recorded for the *L. cossentinii* (16.27 mg kojic acid equivalent (KAE)/g) and 15.65 and 14.48 mg KAE/g for *L. luteus* and *L. albus*, respectively. As far as we know, some phenolics, such as quercetin and kaempferol, inhibit tyrosinase activity due to their ability to chelate the copper in the active site, leading to irreversible inactivation of tyrosinase [32]. According to the literature, α-amylase is a key enzyme in the breakdown of carbohydrates and intestinal absorption [33].

The last enzyme mentioned above is α-amylase, which exhibited a low α-amylase inhibition potential for all the tested extracts—0.16 mmol acarbose equivalent (ACAE)/g for both *L. luteus* and *L. cossentinii* and 0.15 mmol (ACAE)/g for *L. albus.* Regarding a possible comparison between different *Lupinus* species based on the in vitro enzyme inhibition, there is no information available in the literature to make a realistic inference.

### 2.4. Untargeted Metabolomic Profile and Discrimination of the Different Lupinus Species

In this study, we have used an untargeted metabolomic approach (based on UHPLC-ESI/QTOF-MS) to evaluate the phytochemical profile of the methanolic extracts of the three *Lupinus* species, namely *L. albus*, *L. cossentinii* and *L. luteus*. Overall, the approach used allowed to putatively annotate 249 compounds, including polyphenols, alkaloids and other compounds. In addition, the MSMS annotation using QC samples allowed to confirm the structural identity of 118 compounds, that are reported in Supplementary File S1 together with their relative abundance values and composite MS and MSMS spectra. Regarding specific phytochemicals, the UHPLC-QTOF mass spectrometry approach revealed 92 polyphenols (42 flavonoids, 22 tyrosol equivalents, 17 phenolic acids and 3 lignans) and 42 typical alkaloids (such as lupinine, lupanine and epilupinine). The entire list of compounds identified across the different seeds is provided in Supplementary File S1.

**Figure 1:**
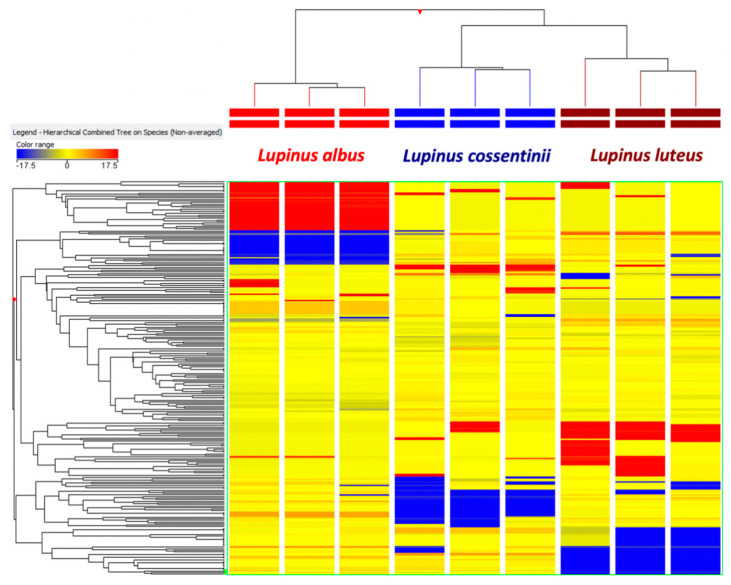
Non-averaged unsupervised hierarchical clustering of the phenolic profiles of *Lupinus albus*, *luteus* and *cossentinii* (similarity: Euclidean; linkage rule: Ward). The compounds’ intensity was used to build up a heat map, on the basis of which the clusters were generated. LA: *L. albus;* LL: *L. luteus;* LC: *L. cossentinii*.

**Figure 2:**
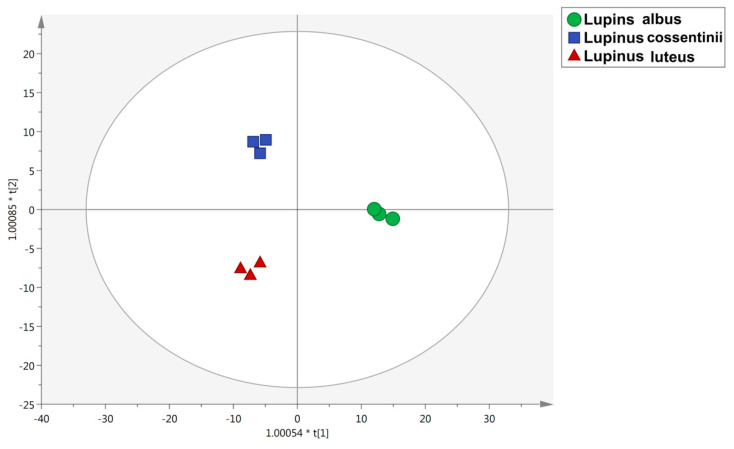
Orthogonal projection to latent structures discriminant analysis (OPLS-DA) built considering the phytochemical profile (from UHPLC-QTOF) of different seeds of the three Lupinus species.

Thereafter, multivariate statistics was used to identify the differences and similarities between the different *Lupinus* species, according to the phytochemical profile detected. The unsupervised HCA is reported in Figure 1. As can be observed, the heat map revealed that *L. albus* was characterized by a definitely different and exclusive phytochemical profile, when compared with *L. luteus* and *L. cossentinii* (there were found to cluster together). Starting from this consideration, the supervised orthogonal projection to latent structures discriminant analysis (OPLS-DA), a multivariate statistical approach, was performed to account for the differences in the phytochemical pattern amongst the different species of *Lupinus* (Figure 2); the loading plot of the model is provided in Supplementary Materials. Afterwards, the variables importance in projection (VIP) analysis was carried out to select those compounds better contributing for discrimination purposes. The discriminating metabolites are shown in Supplementary File S1, grouped according to the chemical class and sub-class they belong to. As reported in Supplementary File S1, 38 phenolic compounds as being able to mainly discriminate each species have been identified.

The phenolic markers with a VIP score > 1 belonged mainly to flavonoids 36.1%, including anthocyanins (cyanidin 3-*O*-xylosyl-rutinoside; cyanidin 3-*O*-glucosyl-rutinoside; cyanidin 3-*O*-sambubiosyl 5-*O*-glucoside; pelargonidin 3-*O*-(6’’-succinyl-glucoside)), flavones (apigenin 6-*C*-glucoside; apigenin 7-*O*-glucoside) and flavonols (these were kaempferol 3-*O*-rhamnoside; quercetin 3-*O*-glucosyl-xyloside; quercetin 4’-*O*-glucoside). The following class in terms of abundance was the phenolic acids (33.3%), with hydroxycinnamic acids, followed by other phenolics (27.7%) and only one lignan (i.e., secoisolariciresinol di-*O* glucoside) as discriminant compound. In particular, the highest VIP scores were observed for carnosic acid (1.29), carvacrol (1.28), thymol (1.28), hydroxycinnamic acid 1,2-Disinapoylgentiobiose (1.29) and for anthocyanin cyanidin 3-*O*-glucosyl-rutinoside (1.25). Overall, each phenolic compound was reported according to its chemical class, together with its VIP score (degree of discrimination). The score plot of OPLS-DA multivariate approach underlined clear diversification among the three species of lupin seeds. Notably, the three *Lupinus* extracts showed evidence of a good separation into the score plot space, thus confirming the discriminatory potential of the phytochemical profiling investigated.

In a previous study, Dueñas et al. [21] confirmed that flavonoids were the most abundant phenolic compounds in the analyzed *L. angustifolius* seeds. Moreover, Stobiecki et al. [34] and Karamac et al. [35] reported a high level of phenolic acids (*p*-coumaric acid derivative) which is consistent with our results. Likewise, Siger et al. [31] claimed that yellow lupin seeds are richer in phenolic acids and flavonoids than the white and narrow-leafed lupin.

### 2.5. Pearson Correlations

In order to investigate the potential correlations between the different phytochemical profiles of *Lupinus* extracts (obtained both from semiquantitative values by UHPLC-QTOF-MS analysis and from spectrophotometric assays) and the in vitro assays of antioxidant activity and enzymatic inhibition properties, Pearson correlation coefficients were used; a summarizing table is reported in Supplementary File S1. Overall, our findings revealed quite good correlations between phenolics and biological properties. In this regard, secondary metabolites (such as polyphenols) are known to be characterized by different mechanisms of radical scavenging and enzymatic inhibitions, thus making them potential agents to treat many ailments, such as cardiovascular diseases, cancer, diabetes and other pathologies [36,37,38,39].

Going into details of the correlations, we found that alkaloids established no positive correlations with the different assays, recording only negative and significant correlation coefficients when considering DPPH, CUPRAC, FRAP and alpha-amylase inhibition.

Additionally, luteolin equivalents were strongly correlated (*p* < 0.01) to CUPRAC and FRAP activities, also establishing a significant correlation with the inhibition of alpha-amylase (0.752; *p* < 0.05). Overall, flavones and their derivatives have been widely described as good scavengers of free radicals with potential pharmaceutical and food applications. The predominant mechanism of the radical-scavenging action of flavones occurs via the donation of a single electron to the radical, thus resulting in the formation of a semiquinone radical [40]. Additionally, it was demonstrated that the electron-donating group of ring B reduce the O-H bond dissociation energy, thus increasing the radical scavenging/antioxidant activity of these compounds. In this regard, flavones lacking the hydroxyl group do not exhibit significant antioxidant activity [40]. Regarding the significant correlations observed between flavones and alpha-amylase inhibition, it is known that flavonoids exhibit a potential inhibition towards amylolytic enzymes. In particular, as reviewed by Giuberti et al. [39], the 2,3-double bond in ring C of flavones is responsible for an electron delocalization between ring C and ring A that promotes the formation of a stable benzopyrone system; this latter is able to bind the indole ring of Trp59 residue, therefore reducing the catalytic activity of the alpha-amylase enzyme. Therefore, our findings demonstrated that the role played by *Lupinus* flavones towards starch digestibility could represent a valid strategy to design functional foods and/or to manage type II diabetes.

Regarding the other phenolic classes, we observed significant correlation coefficients (*p* < 0.05) between lignans with ABTS and BChE inhibitory activities (Supplementary File S1) of 0.691 and 0.716, respectively. Lignans have been recently studied as potential modulators of the gut–brain axis. In addition, the structural similarity between enterolignans and 17β-estradiol allows enterolignans to represent natural ligands of estrogen receptors. In this regard, gut bacterial metabolism is able to convert dietary lignans into therapeutically relevant enterolignans, such as enterolactone and enterodiol. Enterolignans are characterized by various biologic activities, including antioxidant, anti-inflammatory and apoptotic effects [41]. Interestingly, the potential of lignans to inhibit carbonic anhydrase, AChE and BChE, may represent a valid strategy towards neuroprotection, in order to prevent memory loss events (typical of Alzheimer’s-affected patients) [42,43].

In our experimental conditions, we found the highest number of significant correlation coefficients (i.e., 5; Supplementary File S1) for the phenolic group represented by lower-molecular-weight compounds (i.e., tyrosol equivalents). These compounds were found to be significantly correlated to DPPH, CUPRAC and FRAP activities, followed by the inhibition of AChE and alpha-amylase enzymes. Interestingly, untargeted metabolomics revealed a strong matrix effect when considering the class of other phenolics, with LC being particularly abundant in carvacrol, thymol and 5-heptadecylresorcinol, whilst a great abundance of pyrogallol was outlined for the LL species (Supplementary File S1). Among the other phenolics group, we found an abundance of alkylresorcinols. The latter, prevalently described in whole cereal grains, have been reported to inhibit enzyme activity, also preventing bacterial/fungal infections, reducing cholesterol absorption and resisting oxidation [44]. In particular, some in vitro experiments have shown that 5-alkylresorcinol has antioxidant activity, which is mainly due to the two indirect positions of hydroxyl groups on the benzene ring, which can scavenge free radicals and generate protons [44]. Additionally, these phenolics have been reported as natural alpha-glycosidase inhibitors in whole grain foods, successfully reducing fasting blood glucose in obese mice, induced by high-fat and high-sugar diets, increasing glucose tolerance and insulin sensitivity in mice and fecal cholesterol excretion in mice, thus reducing cholesterol concentrations in the blood of these rodents [45]. Stasiuk et al. [46] studied the effect of these compounds (extracted from whole wheat) on the activity of AChE. The authors demonstrated that these phenolic lipids could inhibit the activity of AChE and the effect is strictly dependent on the structural characteristics of its hydrophilic part combined with the alkyl chain length. Therefore, considering that these compounds were particularly abundant in our *Lupinus* extracts, future studies (based, for example, on in silico models) should concentrate on understanding the molecular mechanisms of these inhibitions.

Finally, we also found strong correlation coefficients between the class of phenolic acids and several activities. As can be observed in Supplementary File S1, phenolic acids were highly correlated (*p* < 0.01) with ABTS and phosphomolybdenum activities, also showing the ability to inhibit the enzyme BChE (correlation coefficient: 0.873; *p* < 0.01). In a previous work, Siger et al. [31] investigated the antioxidant activity and phenolic content in three lupin species, namely *L. albus*, *L. luteus*, and *L. angustifolius*. The authors reported a positive and strong correlation between the content of some phenolic acids (mainly protocatechuic acid and *p-*hydroxybenzoic acid) and the antioxidant activity measured by DPPH method. However, in our experimental conditions, the studied *Lupinus* species were mainly abundant in hydroxycinnamic acids, with the highest number of compounds annotated in the cultivar LA, being cinnamoyl glucose and isomers of diferuloylquinic acid the most abundant phenolics (Supplementary File S1). Therefore, when considering potential comparison with scientific literature, not only the genetic diversity but also the impact of pedoclimatic conditions of the profile of secondary metabolites, phenolics, should be considered [47].

## 3. Materials and Methods

### 3.1. Plant Material

The field experiment was carried out in 2018 at the research stations of Tabarka, governorate of Jendouba, Tunisia (36°57′18″ N, 8°45′18″ E), with an average annual rainfall of 1099 mm, and in El Haouaria, governorate of Nabeul, Tunisia (37°01′ N, 10° 55′ E), with an average annual rainfall of about 591 mm. Seeds of three species of *Lupinus* (*L. luteus*, *L. cossentinii* and *L. albus*) were collected in Tunisia. Seeds of *L. luteus* and *L. albus* were collected in Tabarka—*L. luteus* from wild population and *L. albus* from a cultivated field. However, seeds of *L. cossentinii* were collected in El Haouaria. Thus, the collection consisted of about 50 plants per square which must be separated from each other by at least 5 m and at the rate of 3 squares per site. Lupine seeds were harvested as soon as the moisture content reached 14–15%, in order to promote good preservation of the seeds. The harvest took place towards the end of June, as soon as the pod was ripe. Delays can result in significant yield loss due to lodging, pod bursting and pod drop. *Lupinus* from the wild population were authenticated by Zeineb Gammar-Ghrabi (INAT, University of Carthage). Seeds were stored in a laboratory, frozen and then numbered as follows: LA_2018_1-3; LC_2018_4-6; LL_2018_7-9 for *L. albus*, *L. cossentinii* and *L. luteus*, respectively.

### 3.2. Extraction of Phytochemicals and Untargeted Profiling by UHPLC-QTOF-Mass Spectrometry

Seeds of each species of *L. albus*, *L. luteus* and *L. cossentinii* (1 g each) were ground and the flour of each was used to extract aqueous phase in 10 mL of 0.1% formic acid in 80% methanol (LCMS grade, VWR, Milan Italy) using an ULTRA-TURRAX (Ika T25, Staufen, Germany). The extracts were centrifuged at 7000× *g* for 10 min at 4 °C and the supernatants were filtered with 0.22 nm cellulose syringe filters in vials, which were stored at −18 °C until analysis. Thereafter, the phenolic profile of the different methanolic extracts was investigated according to ultra-high-pressure liquid chromatography (UHPLC) coupled quadrupole-time-of-flight mass spectrometry (UHPLC-ESI/QTOF-MS), as previously reported [48]. The chromatographic separation was based on a C18 column (Agilent Zorbax eclipse plus; 50 mm × 2.1 mm, 1.8 μm) and a binary mixture of water and acetonitrile as mobile phases (LC-MS grade, VWR, Milan, Italy), both acidified with 0.1% formic acid (*v*/*v*). A positive full scan acquisition of accurate masses (100–1200 *m*/*z* range) at a rate of 0.8 spectra/s was also used. The injection volume was 6 μL, in triplicate for each sample; the sequence of injection was randomized, and each set of samples was preceded by a blank control (i.e., extraction solvent, aqueous methanol). Quality control samples (QCs) were prepared by pooling an aliquot of each sample extract and injected at the beginning of the sequence and after every 10 sample injections. The QCs were analyzed in data-dependent auto-MS/MS mode using 10 precursors per cycle (1 Hz, 50–1200 *m*/*z*, positive polarity, active exclusion after 2 spectra), with typical collision energies of 10, 20 and 40 eV for collision-induced decomposition. The source conditions were the following: nitrogen was used as both sheath gas (10 L/min and 350 °C) and drying gas (8 L/min and 330 °C), nebulizer pressure was 60 psi, nozzle voltage was 300 V and capillary voltage was 3.5 kV.

Compound identification from raw mass features was made in Profinder B.06 (from Agilent Technologies), according to the ‘find-by-formula’ algorithm [48]. The annotation process considered the isotope pattern (i.e., monoisotopic mass, isotopic spacing and isotopic ratio) and a 5-ppm tolerance for mass accuracy, following both mass and retention time alignments as post-acquisition filters, thus reaching a level 2 of confidence in annotation (i.e., putatively annotated compounds; [49]). To achieve a higher degree of confidence in annotation and structural confirmations, a further identification step was carried out from QCs in MS-DIAL 4.28 [50], using the publicly available MS/MS experimental spectra built into the software (e.g., Mass Bank of North America) and MS-Finder in-silico fragmentation from compounds reported in FoodDB and PlantCyc databases [51]. In addition, for MS-only experiments, the reference database for annotations was produced by merging Phenol-Explorer 3.6 (i.e., a comprehensive database containing more than 500 phenolics) to the relevant lupin-alkaloids reported in literature [52,53].

Finally, the dataset obtained was classified according to each chemical class and subclass annotated and then cumulative abundances for each phenolic class were calculated from calibration curves of pure individual standard solutions (Extrasynthese, Lyon, France; purity > 98%) analyzed under the same conditions [48]. The phenolic standards used were the following: cyanidin (anthocyanins), luteolin (flavones and other flavonoids), quercetin (flavonols), ferulic acid (hydroxycinnamic acids and other phenolic acids), sesamin (lignans) and tyrosol (tyrosols and other remaining phenolics). The total alkaloids were semi-quantified using a calibration curve of sanguinarine (Sigma grade, Sigma-Aldrich, St. Louis, MO, USA). The results were expressed as mg equivalents/g dry weight (dw) in each of the three replicates (*n* = 3).

### 3.3. In Vitro Antioxidant Capacity

Antioxidant protocols included reducing power (cupric reducing antioxidant capacity (CUPRAC) and ferric reducing power (FRAP)), metal chelating, phosphomolybdenum (PBD) and free radical scavenging (2,2-diphenyl-1-picrylhydrazyl (DPPH) and 3-ethylbenzothiazoline-6-sulphonic acid (ABTS)) activities. Trolox and ethylenediaminetetraacetic acid (EDTA) were used as positive controls in the antioxidant assays. Experimental details were as described previously by Grochowski et al. [54].

### 3.4. In Vitro Enzyme Inhibitory Assays

Inhibitory effects of the extracts were tested against different enzymes (tyrosinase, 𝛼-amylase, 𝛼-glucosidase and cholinesterases). Galantamine (for cholinesterase), kojic acid (for tyrosinase) and acarbose (for α-amylase) were used as positive controls and the obtained results were explained as the equivalents of these standard compounds. All experimental procedure were reported in our previous papers [55,56].

### 3.5. Statistical Analysis

Analysis of variance (one-way ANOVA; *p* < 0.05) was performed using PASW Statistics 26.0 (SPSS Inc., Chicago, IL, USA) to check for significant differences in semi-quantitative values of each representative subclass of phenolic compounds, together with in vitro antioxidant and enzymatic inhibitory activities. In addition, Duncan’s post-hoc test was used to identify homogenous subclasses. Finally, Pearson correlation coefficients were finally calculated to highlight significant correlations (*p* < 0.05 and *p* < 0.01; two-tailed) between the phytochemical contents and the different biological activities.

The Agilent Mass Profiler Professional B.06 software was used for filtering and normalization of the metabolomics dataset [48]. In this regard, compound abundance was log_2_ transformed, normalized at 75th percentile and then baselined versus the median of each compound in all samples. Different statistical approaches were used for data modelling, namely, hierarchical cluster analysis (HCA) (as an unsupervised tool), setting the similarity measure as ‘Euclidean’ and ‘Ward’ as the linkage rule, and then orthogonal projection to latent structures discriminant analysis (OPLS-DA) supervised modelling (using the software SIMCA 13, Umetrics, Malmo, Sweden). The model was cross-validated (CV-ANOVA; *p* < 0.01) and inspected for outliers (Hotelling’s T-squared distribution, using 95% and 99% confidence limits for the suspect and strong outliers, respectively) and permutation testing (N = 100) was performed to exclude overfitting. The parameters related to prediction model reliability, namely, prediction ability Q^2^(cum) and degree of correlation R^2^Y(cum) and R^2^X(cum), were recorded. The variable importance in projection (VIP) approach was then used to select the best marker compound of each *Lupinus* species under investigation, considering the variables having the highest discrimination potentials (VIP score > 1). Finally, fold change (FC) analysis was conducted to provide the accumulation trends of each marker compound highlighted by the VIP selection method.

## 4. Conclusions

Lupine, a crop showing high protein and fiber contents, also represents a rich source of bioactive peptides, alkaloids, polyphenols, phytosterols and tocopherols. The significant concentrations of these phytochemicals make lupine flour a potential food ingredient, especially for baked foods. In this work, all studied lupins, i.e., one cultivated *L. albus* and two wild population of *L. luteus* and *L. cossentinii*, exhibited a wide bioactive profile with considerable antioxidant activity. The semi-quantitative analysis of the different phenolic subclasses was performed according to a UHPLC-QTOF-MS approach. The results suggest a need to further study this food matrix in order to better assist breeding programs. In addition, different alkaloid levels were found for *L. albus*, *L. luteus* and *L. cossentinii*, respectively, and this was in agreement with the geographical origin of the species. However, for all lupinus collections, it is important to profile the mineral composition and primary and secondary metabolites in order to select the best species for the benefit of farmers and consumers. All species need further testing to accurately correlate biological activities in order to identify the appropriate species for protein-rich food or feed production.

## Figures and Tables

**Table 1:** Semi-quantification of the different phenolic classes and total alkaloids in the different Lupinus extracts.

Class	*L. albus*	*L. cossentinii*	*L. luteus*
Anthocyanins	0.11 ± 0.01 ^b^	0.13 ± 0.03 ^b^	0.26 ± 0.08 ^a^
Other flavonoids	0.06 ± 0.005	0.13 ± 0.08	0.09 ± 0.02
Flavonols	0.29 ± 0.02	0.31 ± 0.13	0.30 ± 0.22
Lignans	0.26 ± 0.04 ^a^	0.04 ± 0.02 ^b^	0.10 ± 0.01 ^b^
Other phenolics	0.27 ± 0.03 ^b^	5.82 ± 0.47 ^a^	0.79 ± 0.14 ^b^
Phenolic acids	0.15 ± 0.06	0.11 ± 0.04	0.06 ± 0.01
Total alkaloids	4.42 ± 0.26 ^a^	0.84 ± 0.39 ^b^	3.66 ± 0.73 ^a^

Data are presented as mean values (mg/g DW) ± standard deviation (*n* = 3) and multiple comparisons (ANOVA) were performed using Duncan’s post hoc test. Different superscript letters within each row indicate significant differences (*p* < 0.05), whilst no superscript letters indicate no significant differences (*p* > 0.05).

**Table 2:** In vitro antioxidant capacity in seeds of three Tunisian lupin species.

Sample	DPPH (mg TE/g)	ABTS (mg TE/g)	CUPRAC (mg TE/g)	FRAP (mg TE/g)	Phosphomolybdenum (mmol TE/g)	Metal Chelating (mg EDTAE/g)
*L. albus*	1.92 ± 0.05 ^b^	17.58 ± 2.51 ^a^	7.20 ± 0.62 ^b^	4.44 ± 0.28 ^ab^	0.47 ± 0.03 ^a^	3.38 ± 0.04 ^b^
*L. cossentinii*	3.50 ± 0.22 ^a^	10.17 ± 1.70 ^b^	8.95 ± 1.16 ^a^	5.75 ± 0.65 ^a^	0.44 ± 0.06 ^a^	3.55 ± 0.53 ^b^
*L. luteus*	0.39 ± 0.04 ^c^	4.67 ± 0.42 ^c^	7.51 ± 0.07 ^ab^	4.11 ± 0.04 ^b^	0.41 ± 0.07 ^a^	4.68 ± 0.62 ^a^

Data are presented as mean values ± standard deviation (*n* = 3) and multiple comparisons (ANOVA) were performed using Duncan’s post hoc test. The same superscript letters within each column indicate the absence of a significant difference.

**Table 3:** Inhibitory activity of enzymes in seeds of three Tunisian lupin species.

Sample	ACHE Inhibition (mg GALAE/g)	BCHE Inhibition (mg GALAE/g)	Tyrosinase Inhibition (mg KAE/g)	Amylase Inhibition (mmol ACAE/g)
*L. albus*	0.90 ± 0.09 ^a^	1.74 ± 0.07 ^a^	14.48 ± 0.28 ^b^	0.15 ± 0.01 ^a^
*L. cossentinii*	1.04 ± 0.02 ^a^	0.99 ± 0.08 ^b^	16.27 ± 0.46 ^a^	0.16 ± 0.01 ^a^
*L. luteus*	0.93 ± 0.06 ^a^	0.47 ± 0.10 ^c^	15.65 ± 0.74 ^a^	0.16 ± 0.01 ^a^

Data are presented as mean values ± standard deviation (*n* = 3) and multiple comparisons (ANOVA) were performed using Duncan’s post hoc test. The same superscript letters within each column indicate the absence of a significant difference.

## Data Availability

The data presented in this study are available in Supplementary Materials (Supplementary File S1).

## Supplementary Materials

The following are available online. Supplementary File S1: Metabolomic dataset from UHPLC-QTOF-MS analysis, together with a list of VIP discriminant metabolites and a loading plot following OPLS-DA. Supplementary File S2: The Pearson correlation coefficients between phytochemicals composition and the in vitro biological activities are also provided.

## Sample Availability

Samples of the compounds are not available from the authors.
